# Study of Periodontal Health in Almada-Seixal (SoPHiAS): a cross-sectional study in the Lisbon Metropolitan Area

**DOI:** 10.1038/s41598-019-52116-6

**Published:** 2019-10-29

**Authors:** João Botelho, Vanessa Machado, Luís Proença, Ricardo Alves, Maria Alzira Cavacas, Luís Amaro, José João Mendes

**Affiliations:** 1 Periodontology Department, Egas Moniz Dental Clinic (EMDC), Egas Moniz, CRL, Monte de Caparica, Portugal; 2 Clinical Research Unit (CRU), Centro de Investigação Interdisciplinar Egas Moniz (CiiEM), Egas Moniz, CRL, Monte de Caparica, Portugal; 3 Quantitative Methods for Health Research Unit (MQIS), CiiEM, Egas Moniz, CRL, Monte de Caparica, Portugal; 4 Health Centers grouping (HCG) Almada-Seixal, Regional Health Administration of Lisbon and Tagus Valley (RHALTV), Lisbon, Portugal; 5 Hospital Garcia de Orta, Almada, Portugal

**Keywords:** Risk factors, Dental diseases, Chronic inflammation

## Abstract

This study aimed to describe the prevalence and extent of periodontal diseases among adults in the southern region of the Lisbon Metropolitan Area. This population-based cross-sectional study included 1,064 randomized participants (aged 18 to 95 years, 617 females/447 males). Sociodemographic, behaviours and medical information were recorded. Periodontal conditions were assessed with a full-mouth circumferential periodontal examination. It was used the American Association of Periodontology/European Federation of Periodontology 2017 case definitions. A logistic regression analysis was applied to ascertain hypothetical risk factors towards periodontitis. The prevalence of periodontitis was 59.9%, with 24.0% and 22.2% of the participants exhibiting severe and moderate periodontitis, respectively. The risk of periodontitis significantly increased with age (OR = 1.05, 95% CI: 1.04–1.06), for active and former smokers (OR = 3.76 and OR = 2.11, respectively), with lower education levels (OR = 2.08, OR = 1.86, for middle and elementary education, respectively) and with diabetes mellitus (OR = 1.53). This study confirms a high burden of periodontitis in the target (Portuguese) sub-population. The findings provide a comprehensive understanding that will empower appropriate national public oral health programmes and population-based preventive actions.

## Introduction

Prevalence of periodontal diseases endures a substantial epidemiological challenge, while estimates presented in recent years have been very dissimilar, even in countries with alike socio-economically standards^1–3^. Thus, these have contributed to the lack of comprehensive understanding of the periodontal status worldwide. In addition, periodontitis has a large socioeconomic impact and it is estimated that is responsible for 54 billion USD/year in lost productivity and a major portion of the 442 billion USD/year cost for oral diseases^4^. Also, these polymicrobial inflammatory diseases are extremely impacting on other systemic conditions, such as diabetes mellitus (DM)^5^, stroke^6,7^, rheumatoid arthritis^8^, inflammatory bowel disease^9^, or preterm birth^10^.

Over the last decades, periodontitis case definitions have undergone paradigmatic changes evolving from a diagnosis based in terms of clinical attachment loss (CAL) and probing depth (PD), as proposed by the CDC Working Group^11^ and revised accordingly^12^, to a diagnosis proposed in the new American Association of Periodontology (AAP)/European Federation of Periodontology (EFP) based mainly upon CAL and considering the interproximal space as an adjacent common zone^13^. In fact, all efforts made to improve these diagnostic criteria focused on the prevention of underestimation of periodontitis and to reveal the natural history of periodontitis, especially in older subjects.

To date, very few data have provided a comprehensive assessment of the periodontal status of the Portuguese population^14–16^. A single national epidemiological study was conducted, in 2015, by the Portuguese Health General Directorate^15^ using the Community Periodontal Index of Treatment Needs (CPITN). The obtained results estimated a prevalence of 10.8% and 15.3% of periodontal diseases in adults and elderly, respectively^15^. These results contrast, specifically, and due to geographical proximity, with the last national Spanish periodontal survey where 38.4% of subjects had periodontal pockets^17^, as well with other developed countries studies where found prevalence ranged from 51.0 to 88.3% in the USA, Italy (Turin), Norway (Troms) or Germany (Pomerania)^12,18–21^ and World Health Organization (WHO) global reports^3^.

Due to the recent disclosure of the new periodontal stage consensus^13^, there is still limited data coming from epidemiological studies employing these diagnostic criteria in Europe. Also, the available Portuguese national epidemiologic data relies on CPITN methodology which is inadequate to describe the periodontal status of populations^22^. Consequently, it is essential to carry out studies using the new case definitions which will allow a comprehensive understanding of the current periodontal status in the Portuguese population and the assessment of associated risk factors, to allow future international comparability. Therefore, it is mandatory to investigate the burden of periodontal disease of a Portuguese target population and its associated risk factors, to serve as a foundation for future national public health strategies.

Therefore, this study was aimed to investigate the distribution of periodontal diseases using a population-based stratified sample of adults from the southern region of the Lisbon Metropolitan Area. The prime purposes of this study were: (1) to comprehensively describe the prevalence and extent of periodontal diseases according to the Workshop in 2017^13^, (2) to evaluate potential periodontal diseases risk indicators.

## Results

### Study sample

The characteristics of the 1,064 subjects included in the study, according to the periodontal diagnosis, are shown in Table 1. The mean age of participants was 60.9 (±16.3) years, 58.0% were women, 63.3% reported having an elementary education level and 52.2% were retired. The prevalence of moderate and severe periodontitis increased with age. Moreover, the majority of the population (81.9%) report not knowing what periodontal disease is, 37.6% brush their teeth once or less daily, and 70.2% of subjects with severe periodontitis have never performed interproximal cleaning.

**Table 1:** Sociodemographic characteristics, behaviors, attitudes towards oral health and medical information (diabetes and comorbidity) of the included participants, presented as n (%), according to the severity of periodontal status (N = 1,064).

	No Disease n (%)	Gingivitis n (%)	Mild n (%)	Moderate n (%)	Severe n (%)	Total n (%)
**Gender**						
Male	117 (34.2)	23 (27.1)	64 (43.8)	111 (47.0)	132 (51.8)	447 (42.0)
Female	225 (65.8)	62 (72.9)	82 (56.2)	125 (53.0)	123 (48.2)	617 (58.0)
**Age (years)**						
18–30	34 (9.9)	17 (20.0)	10 (6.8)	1 (0.4)	0 (0.0)	62 (5.8)
31–40	42 (12.3)	7 (8.2)	11 (7.5)	10 (4.2)	5 (2.0)	75 (7.1)
41–50	62 (18.1)	11 (12.9)	21 (14.4)	23 (9.8)	19 (7.4)	136 (12.8)
51–60	50 (14.6)	5 (5.9)	15 (10.3)	32 (13.6)	35 (13.7)	137 (12.9)
61–70	82 (24.0)	26 (30.6)	45 (30.8)	81 (34.3)	94 (36.9)	328 (30.8)
71–80	58 (17.0)	16 (18.8)	38 (26.0)	57 (24.2)	75 (29.4)	244 (22.9)
>80	14 (4.1)	3 (3.5)	6 (4.1)	32 (13.6)	27 (10.6)	82 (7.7)
**Educational level**						
No education	8 (2.3)	3 (3.5)	6 (4.1)	11 (4.7)	14 (5.5)	42 (4.0)
Elementary	176 (51.5)	50 (58.8)	94 (64.4)	173 (73.3)	180 (70.6)	673 (63.3)
Middle	94 (27.5)	23 (27.1)	35 (24.0)	38 (16.1)	43 (16.9)	233 (21.9)
Higher	64 (18.7)	9 (10.6)	11 (7.5)	14 (5.9)	18 (7.1)	116 (10.9)
**Marital status**						
Single	81 (23.7)	23 (27.1)	22 (15.1)	22 (9.3)	22 (8.6)	170 (16.0)
Married/Union of fact	213 (62.3)	49 (57.7)	92 (63.0)	158 (66.9)	172 (67.5)	684 (64.3)
Divorced	25 (7.3)	8 (9.4)	20 (13.7)	25 (10.6)	25 (9.8)	103 (9.7)
Widowed	23 (6.7)	5 (5.9)	12 (8.2)	31 (13.1)	36 (14.1)	107 (10.1)
**Occupation**						
Student	11 (3.2)	7 (8.2)	1 (0.7)	0 (0.0)	0 (0.0)	19 (1.8)
Employed	138 (40.4)	27 (31.8)	53 (36.3)	52 (22.0)	57 (22.3)	327 (30.7)
Unemployed	63 (18.4)	16 (18.8)	13 (8.9)	35 (14.8)	36 (14.1)	163 (15.3)
Retired	130 (38.0)	35 (41.2)	79 (54.1)	149 (63.1)	162 (63.5)	555 (52.2)
**Smoking status**						
Non-smoker	238 (69.6)	58 (68.2)	88 (60.3)	122 (51.7)	120 (47.1)	626 (58.8)
Former smoker	71 (6.7)	14 (16.5)	38 (26.0)	76 (32.2)	94 (36.9)	293 (27.5)
Current Smoker	33 (3.1)	13 (15.3)	20 (13.7)	38 (16.1)	41 (16.1)	145 (13.6)
**Family income (monthly**, **€)**						
<=600	74 (22.0)	24 (30.0)	37 (25.5)	61 (26.2)	74 (29.3)	270 (25.8)
601–1,500	194 (57.7)	46 (57.5)	92 (63.4)	137 (58.8)	143 (56.5)	612 (58.4)
>1,500	68 (20.2)	10 (12.5)	16 (11.0)	35 (15.0)	36 (14.2)	165 (15.8)
**Periodontal diseases awareness**						
Yes	75 (21.9)	12 (14.1)	26 (17.8)	36 (15.2)	44 (17.3)	193 (18.1)
No	267 (78.1)	73 (85.9)	120 (82.2)	200 (84.8)	211 (82.7)	871 (81.9)
**Brushing frequency (daily)**						
3+	65 (19.0)	16 (18.8)	24 (16.4)	39 (16.5)	27 (10.6)	171 (16.1)
2	196 (57.3)	36 (42.3)	74 (50.7)	122 (51.7)	132 (51.8)	560 (52.6)
1	78 (22.8)	29 (34.1)	46 (31.5)	65 (27.5)	84 (32.9)	302 (28.4)
0	3 (0.9)	4 (4.7)	2 (1.4)	10 (4.2)	12 (4.7)	31 (2.9)
**Interproximal cleaning**						
Yes	82 (24.0)	10 (11.8)	31 (21.2)	36 (15.3)	26 (10.2)	185 (17.4)
Occasionally	71 (20.8)	10 (11.8)	25 (17.1)	27 (11.4)	28 (11.0)	161 (15.1)
No	189 (55.3)	65 (76.5)	90 (61.6)	173 (73.3)	201 (78.8)	718 (67.5)
**Diabetes Mellitus**						
Yes	44 (12.9)	9 (10.6)	31 (21.2)	46 (19.5)	74 (29.0)	204 (19.2)
No	298 (87.1)	76 (89.4)	115 (78.8)	190 (80.5)	181 (71.0)	860 (80.8)
**Comorbidity (by Aimetti 2015)**						
Yes	260 (76.0)	63 (74.1)	123 (84.2)	206 (87.3)	227 (89.0)	879 (82.6)
No	82 (24.0)	22 (25.9)	23 (15.8)	30 (12.7)	28 (11.0)	185 (17.4)
Total	342 (32.1)	85 (8.0)	146 (13.7)	236 (22.2)	255 (24.0)	1064 (100.0)

### Prevalence and severity of periodontal disease

**Figure 1:**
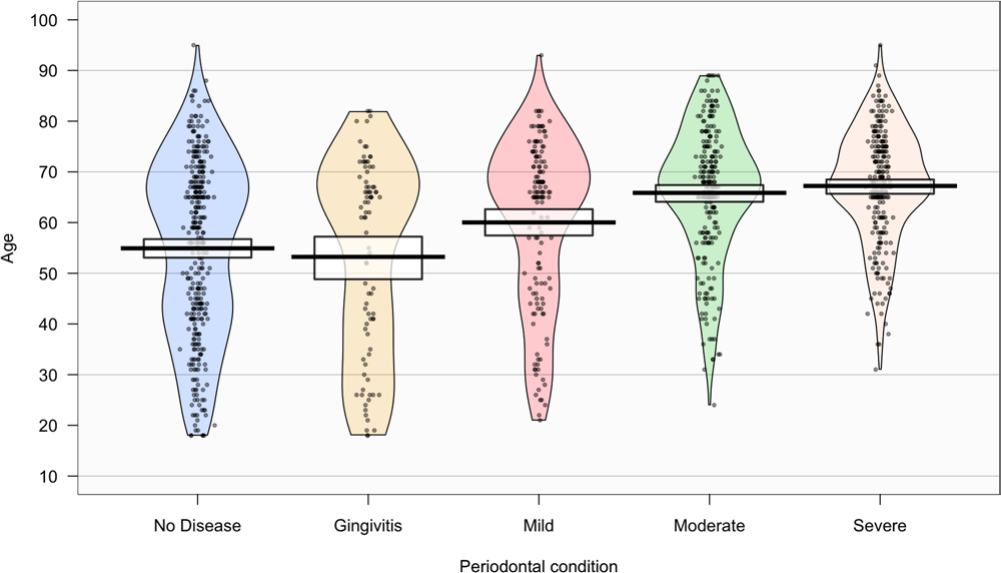
Density plot exhibiting the distribution of periodontal conditions over the age range.

The prevalence of periodontitis (mild, moderate and severe stages) was 59.9% (95% CI 56.9–62.8), with 22.2% of moderate stage (95% CI 19.7–24.8%) and 24.0% of severe stage (95% CI 21.4–26.6%), respectively. The prevalence and correspondent estimates of localized and generalized periodontitis amounted to 23.2% (95% CI: 20.7–25.9%) and 36.7% (95% CI: 33.8–39.6%) respectively (Table 2). Further, periodontal health is a well distributed status, whereas periodontal diseases exhibited a distinct scattering (Fig. 1).

**Table 2:** Prevalence of localized and generalized periodontitis, stratified by age and gender.

	Females		Males		Total	
	n	Prev. (95% CI) (%)	n	Prev. (95% CI) (%)	n	Prev. (95% CI) (%)
**Localized**	**139**	**56.3 (50.1–62.5)**	**108**	**43.7 (37.5–49.9)**	**247**	**23.2 (20.7–25.9)**
18–30	5	3.6 (0.5–6.7)	6	5.6 (1.2–9.9)	11	4.5 (1.9–7.0)
31–40	14	10.1 (5.1–15.1)	5	4.6 (0.7–8.6)	19	7.7 (4.4–11.0)
41–50	20	14.4 (8.6–20.2)	10	9.3 (3.8–14.7)	30	12.1 (8.1–16.2)
51–60	20	14.4 (8.6–20.2)	13	12.0 (5.9–18.2)	33	13.4 (9.1–17.6)
61–70	51	36.7 (28.7–44.7)	34	31.5 (22.7–40.2)	85	34.4 (28.5–40.3)
71–80	25	18.0 (11.6–24.4)	28	25.9 (17.7–34.2)	53	21.5 (16.3–26.6)
80+	4	2.9 (0.1–5.7)	12	11.1 (5.2–17)	16	6.5 (3.4–9.5)
**Generalized**	**191**	**49.0 (44.0–54.0)**	**199**	**51.0 (46.0–56.0)**	**390**	**36.7 (33.8–39.6)**
18–30	0	0.0 (0.0–0.0)	0	0.0 (0.0–0.0	0	0.0 (0.0–0.0
31–40	5	3.6 (1–6.2)	2	1.9 (0.0–3.7)	7	1.8 (0.5–3.1)
41–50	13	9.4 (5.2–13.5)	20	18.5 (13.1–23.9)	33	8.5 (5.7–11.2)
51–60	23	16.5 (11.3–21.8)	26	24.1 (18.1–30.0)	49	12.6 (9.3–15.9)
61–70	71	51.1 (44–58.2)	64	59.3 (52.4–66.1)	135	34.6 (29.9–39.3)
71–80	57	41. (34–48)	60	55.6 (48.7–62.5)	117	30.0 (25.5–34.5)
80+	22	15.8 (10.7–21)	27	25. (19–31.0)	49	12.6 (9.3–15.9)
**Molar–Incisor Pattern**	**330**	**—**	**307**	**—**	**637**	**—**
No	311	94.2 (91.7–96.8)	295	96.1 (93.9–98.3)	606	95.1 (93.5–96.8)
Yes	19	5.8 (3.2–8.3)	12	3.9 (1.7–6.1)	31	4.9 (3.2–6.5)

### Clinical attachment loss (CAL) and probing depth (PD)

**Figure 2:**
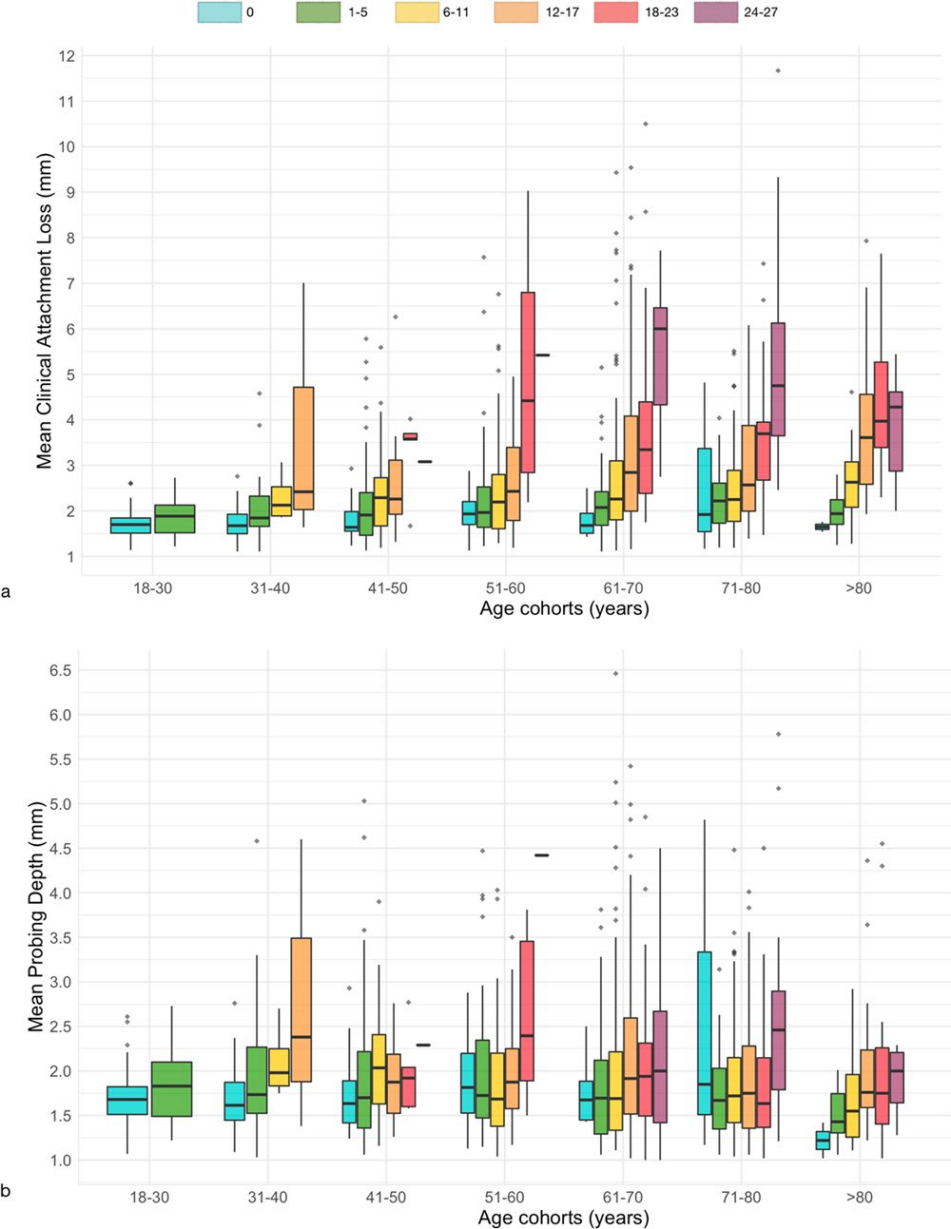
Distribution of tooth loss (coloring) as a function of mean clinical attachment loss (**a**) or mean probing depth (**b**), according to age cohorts (x-axis).

The mean values of PD, CAL, recession (REC), missing teeth and teeth with mobility as well the prevalence and extent of CAL and PD by selected threshold are presented in Table 3. Mean PD and the number of sites with PD ≥4 mm and ≥6 mm remained similar across all age groups. The average CAL and number of sites with CAL ≥4 mm and ≥6 mm were unequally distributed in the population for all age groups, increasing with age, while exhibiting a moderate significant correlation. The number of missing teeth is also related to the mean CAL across age groups, that is, the higher the number of missing teeth the greater the CAL average but for PD this is not so evident (Fig. 2). Mean REC, missing teeth and teeth with mobility also increased with age increase.

**Table 3:** PD, CAL, REC, missing teeth and teeth with mobility (presented as mean, standard deviation and 95% CI for mean), stratified by CAL and PD thresholds (%) (≥4 and ≥6 mm) and age group.

Measures	18–30	31–40	41–50	51–60	61–70	71–80	>80	rho* *(p*-value)	Total
	Mean (SD) [95% CI]	Mean (SD) [95% CI]	Mean (SD) [95% CI]	Mean (SD) [95% CI]	Mean (SD) [95% CI]	Mean (SD) [95% CI]	Mean (SD) [95% CI]		Mean (SD) [95% CI]
Mean PD (mm)	1.8 (0.4) [1.7–1.9]	1.9 (0.6) [1.7–2.0]	1.9 (0.7) [1.8–2.1]	2.0 (0.8) [1.9–2.1]	2.0 (0.9) [1.9–2.1]	1.9 (0.7) [1.8–2.0]	1.9 (0.7) [1.7–2.0]	−0.027 (0.382)	1.9 (0.8) [1.9–2.0]
PD ≥ 4 mm (%)	3.8 (5.4) [1.4–4.1]	6.3 (13.6) [3.2–9.5]	7.9 (13.8) [5.5–10.2]	9.5 (15.5) [6.9–12.1]	9.5 (17.4) [7.7–11.4]	7.6 (14.3) [5.8–9.4]	6.4 (13.6) [3.4–9.4]	0.015 (0.614)	8.0 (15.0) [7.1–8.9]
PD ≥ 6 mm (%)	0.0 (0.2) [0.0–0.1]	1.0 (4.2) [0.1–2.0]	1.3 (4.4) [0.5–2.0]	1.6 (3.9) [1.0–2.3]	2.2 (6.5) [1.5–2.9]	1.8 (6.1) [1.1–2.6]	0.9 (3.1) [0.3–1.6]	0.047 (0.126)	1.6 (5.3) [1.3–1.9]
Mean CAL (mm)	1.8 (0.4) [1.7–1.9]	2.0 (0.8) [1.8–2.2]	2.2 (1.0) [2.1–2.4]	2.6 (1.5) [2.4–2.9]	2.9 (1.6) [2.8–3.1]	2.9 (1.4) [2.8–3.1]	3.4 (1.5) [3.1–3.7]	0.349 (<0.001)	2.7 (1.4) [2.6–2.8]
CAL ≥ 4 mm (%)	3.1 (5.5) [1.7–4.5]	7.9 (15.9) [4.2–11.5]	14.8 (21.0) [11.2–18.4]	22.4 (26.7) [17.9–26.9]	27.9 (28.7) [24.8–31.0]	30.1 (27.3) [26.6–33.5]	40.4 (30.8) [33.7–47.2]	0.416 (<0.001)	24.1 (27.4) [22.5–25.8]
CAL ≥ 6 mm (%)	0.1 (0.2) [0.0–0.1]	2.1 (9.5) [0.0–4.2]	4.0 (10.7) [2.2–5.8]	8.9 (18.0) [5.9–12.0]	12.1 (22.0) [9.7–13.5]	10.9 (18.4) [8.6–13.2]	15.0 (20.7) [10.4–19.5]	0.336 (<0.001)	9.2 (18.4) [8.1–10.3]
Mean REC (mm)	0.0 (0.0) [0.0–0.0]	0.1 (0.3) [0.0–0.2]	0.3 (0.5) [0.2–0.4]	0.7 (0.9) [0.5–0.8]	1.0 (1.1) [0.8–1.1]	1.1 (1.1) [0.9–1.2]	1.6 (1.2) [1.3–1.8]	0.562 (<0.001)	0.8 (1.0) [0.7.0.8]
Missing Teeth (n)	0.9 (1.2) [0.6–1.2]	2.3 (3.3) [1.5–3.0]	5.1 (5.1) [4.2–6.0]	8.2 (5.5) [7.2–9.1]	10.8 (6.5) [10.0–11.5]	12.0 (6.6) [11.2–12.8]	14.0 (7.5) [12.3–15.6]	0.544 (<0.001)	9.1 (7.0) [8.6–9.5]
Teeth with mobility (n)	0.1 (0.4) [0.0–0.2]	0.3 (1.4) [0.0–0.6]	0.6 (1.5) [0.3–0.8]	1.3 (2.5) [0.9–1.8]	1.4 (2.6) [1.1–1.7]	1.1 (1.9) [0.9–1.4]	1.3 (2.2) [0.8–1.8]	0.197 (<0.001)	1.1 (2.2) [0.9–1.2]

*Overall trend across age groups assessed by Spearman’s rank correlation coefficient (rho). Significant correlations identified in bold (p < 0.05).

### Bleeding on probing (BoP) and plaque index (PI)

The mean values of BoP and PI, stratified by periodontitis severity and age group, are presented in Table 4. BoP was equally distributed in the population for all age groups, and increased with level of severity of periodontitis, with a mean of 5.7% for persons with no periodontitis, 15.9% for persons with non-severe periodontitis and 28.5% for persons with severe periodontitis. Similarly, the average PI was 23.2%, and increased with the severity of periodontitis and age.

**Table 4:** Mean Bleeding on Probing (BoP) and Plaque Index (PI) (%) (presented as mean, standard deviation and 95% CI for mean), stratified by periodontitis severity and age group.

Measures	18–30	31–40	41–50	51–60	61–70	71–80	>80	rho* (p-value)	Total
	Mean (SD) [95% CI]	Mean (SD) [95% CI]	Mean (SD) [95% CI]	Mean (SD) [95% CI]	Mean (SD) [95% CI]	Mean (SD) [95% CI]	Mean (SD) [95% CI]		Mean (SD) [95% CI]
Mean BoP (%)	13.6 (17.1) [9.3–18.9]	12.5 (16.8) [8.7–16.4]	15.1 (20.0) [11.7–18.5]	15.7 (22.5) [11.9–19.5]	15.2 (21.3) [12.9–17.5]	14.9 (20.9) [12.2–17.5]	14.3 (20.1) [9.9–18.8]	−0.021 (0.502)	14.8 (20.6) [13.6.16.1]
No Periodontitis	8.3 (9.8) [5.6–11.1]	5.6 (8.3) [3.2–8.0]	5.4 (9.6) [3.1–7.6]	2.9 (4.5) [1.7–4.1]	6.1 (10.8) [4.0–8.1]	5.5 (8.3) [3.6–7.5]	5.8 (13.5) [–1.2-12.7]	**−0.096 (0.048)**	5.7 (9.3) [4.8–6.5]
Non-severe Periodontitis	38.1 (22.4) [23.1–53.1]	23.5 (19.7) [14.5–32.5]	20.2 (16.8) [15.1–25.3]	17.8 (17.9) [12.6–23.1]	13.5 (18.8) [10.1–16.8]	13.8 (20.9) [9.6–18.1]	11.3 (17.5) [5.5–17.0]	**−0.287 (<0.001)**	15.9 (19.6) [13.9–17.9]
Severe Periodontitis	—	34.2 (25.0) [3.1–65.3]	40.5 (28.5) [26.8–54.3]	33.1 (31.4) [22.3–43.9]	27.9 (26.7) [22.4–33.4]	25.4 (24.8) [19.7–31.1]	24.0 (23.5) [14.7–33.3]	**0.127 (0.043)**	28.5 (26.8) [25.2–31.9]
Total Periodontitis	38.1 (22.4) [23.1–53.1]	25.6 (20.7) [17.2–33.9]	26.3 (22.8) [20.6–32.1]	24.3 (25.5) [18.7–30.0]	19.6 (23.6) [16.5–22.8]	18.9 (23.4) [15.4–22.5]	16.6 (13.5) [11.4–21.8]	**−0.181 (<0.001)**	21.0 (23.5) [19.1–22.8]
Mean PI (%)	11.0 (15.9) [7.0–15.0]	10.6 (21.0) [5.7–15.4]	11.4 (19.8) [8.1–14.8]	20.3 (24.4) [15.3–25.3]	23.7 (29.9) [20.4–26.9]	31.1 (32.9) [27.0–35.3]	42.6 (37.6) [34.3–50.8]	**0.296 (<0.001)**	23.2 (30.3) [21.4–25.0]
No Periodontitis	9.5 (14.8) [5.4–13.7]	7.3 (15.0) [3.0–11.6]	6.4 (14.2) [3.0–9.7]	7.7 (13.9) [3.9–11.4]	13.6 (22.7) [9.3–17.9]	20.9 (26.2) [14.9–27.0]	29.3 (36.7) [10.4–48.2]	**0.220 (<0.001)**	12.3 (21.1) [10.3–14.3]
Non-severe Periodontitis	17.9 (20.0) [4.5–31.3]	17.7 (31.3) [3.4–32.0]	16.4 (23.1) [9.3–23.4]	17.0 (22.5) [10.4–23.5]	24.2 (29.1) [19.1–29.3]	33.5 (34.8) [26.4–40.5]	42.1 (38.0) [29.6–54.6]	**0.231 (<0.001)**	26.0 (31.1) [22.8–29.1]
Severe Periodontitis	—	12.7 (13.8) [−4.4–29.7]	19.3 (25.2) [7.2–31.5]	44.7 (39.8) [31.0–58.4]	34.6 (34.2) [27.6–41.6]	38.2 (34.2) [30.3–46.0]	51.7 (36.1) [37.4–65.9]	**0.191 (0.002)**	37.3 (35.1) [33.0–41.6]
Total Periodontitis	17.9 (20.0) [4.5–31.3]	16.7 (28.6) [5.2–28.3]	17.3 (23.6) [11.4–23.2]	28.8 (33.8) [21.4–36.2]	28.7 (31.7) [24.4–32.9]	35.5 (34.5) [30.3–40.8]	46.1 (37.3) [36.8–55.3]	**0.231 (<0.001)**	30.5 (33.2) [27.9–33.1]

BoP – Bleeding on Probing, PI – Plaque Index, SD – Standard Deviation.

*Overall trend across age groups assessed by Spearman’s rank correlation coefficient (rho). Significant correlations identified in bold (p < 0.05).

### Risk factors for periodontitis

Crude and adjusted Odds Ratio (OR) for putative risk factors towards periodontitis were determined and are presented in Table S2 (Supplemental) and Table 5, respectively. Within the final reduced model obtained by a multivariate logistic regression procedure, age (OR = 1.05, 95% CI: 1.04–1.06), educational level (OR = 2.08, 95% CI: 1.32–3.27, OR = 1.86, 95% CI: 1.13–3.05, for middle and elementary education, respectively), smoking status (OR = 3.76, 95% CI: 2.44–5.80 and OR = 2.11, 95% CI: 1.52–2.91, for current smoker and former smoker, respectively) and DM (OR = 1.53, 95% CI: 1.06–2.21) were the significantly risk indicators that were identified towards periodontitis.

**Table 5:** Multivariate logistic regression analysis (final reduced model) (*) on potential risk factors towards periodontitis.

Predictor variables	Odds Ratio (OR)	OR (95% CI)	*p*-value
**Age**	1.05	1.04–1.06	<0.001
**Education**			
Higher	1	—	—
Middle	2.08	1.32–3.27	0.002
Elementary	1.86	1.13–3.05	0.015
No education	2.08	0.88–4.90	0.095
Smoking status	—	—	<0.001
Non-smoker	1	—	—
Current Smoker	3.76	2.44–5.80	<0.001
Former smoker	2.11	1.52–2.91	<0.001
**Diabetes Mellitus**			
No	1	—	—
Yes	1.53	1.06–2.21	0.023

*The model was statistically significant, *χ*2(7) = 174.786, *p* < 0.001, explained 20.5% (Nagelkerke *R*^2^) of the variance and correctly classified 68.7% of cases.

## Discussion

This is the first periodontal population-based representative study carried out in Portugal and one of the very first to use the new periodontitis and gingivitis case definitions^13,23^. The results of this epidemiological study indicate that seven out of ten adults in the target population had some type of periodontal disease, and six out of ten had periodontitis. Moreover, almost half of the population exhibited moderate and severe periodontitis.

In particular, this study sample was low educated, with the majority being below secondary education, and a largest share were under a situation of work inactivity. Also, they self-reported good brushing frequency, poor interproximal cleaning habits and low periodontal disease awareness, being equivalent to the national average^24^. Regarding the systemic state, a very high percentage presented comorbidities. The prevalence of smokers and former smokers were 13.6% and 27.5%, respectively. The DM prevalence was slightly above the national average, however it is explained by the greater percentage of elderly among the included sample^25^.

In the Portuguese panorama, oral health care is mostly provided in private (liberal) practices^26^. Further, there is a lack of dental services in public hospitals and health centres from the National Health Service, and the absence of domiciliary dental care^26^. Besides, the Portuguese Public Oral Health Programme (PPOHP) has only started in 2005 and, in 2008, limited “dental vouchers” were created focusing children, adolescents and vulnerable groups (pregnant, Human Immunodeficiency Virus patients and elders with low-socioeconomic status)^27^. Besides, the serious financial constraints from the 2007–2008 financial crisis have limited access to private dental services. Thus, these reasons might have contributed to the high prevalence of periodontal disease reported in the present study, and support the necessity of a comprehensive national oral program, greater availability of dental services and increased awareness for these diseases.

In Portugal, to date, there is only one national epidemiological study on the prevalence of periodontal disease, though it can not be compared with the present study because it used CPITN methodology^15^. Oppositely, the present findings indicated a higher severity of periodontal destruction. In fact, the use of partial recording protocols underestimate the periodontal prevalence and extent by almost 50%^22,28^.

Few periodontal epidemiological studies provide comprehensive and comparable information in Europe. Furthermore, due to the novelty of the AAP/EFP consensus, the number of studies using this case definition is still scarce. When compared to other European population-based representative studies, the Tromstannen–Oral Health in Northern Norway (TOHNN) study reported an overall prevalence of 9.1% of severe periodontitis^21^, and in the Periodontitis and Its Relation to Coronary Artery Disease (PAROKRANK) in Sweden the prevalence of severe periodontitis was of 6.2%^29^. The Study of Health in Pomerania (SHIP) revealed a prevalence of 17.6% of severe periodontitis and 25.3% of moderate periodontitis^30^, while for the Turin regional survey these prevalence were of 39.9% and 40.8% for severe and moderate periodontitis respectively^18^. In the USA, the National Health and Nutrition Examination Survey (NHANES) 2009–2012 estimates of severe and moderate periodontitis were of 8.9% and 30.9%, respectively^12^.

Drawing parallels with the findings of the present investigation, the prevalence of severe periodontitis was only surpassed by the Turin study, whereas for moderate periodontitis the estimates ranked lower^18^. Notwithstanding, the aforementioned studies used the CDC/AAP case definition as already mentioned, and it is not known what is the difference magnitude between these two classifications.

Undoubtedly, the prevalence of moderate and severe periodontitis peaked in the age of 61–70 years old (34.3% and 36.9%, respectively), having subsequently reduced. Another important aspect to be addressed is the relevantly high prevalence of both localized (34.4%) and generalized (34.6%) periodontitis in the same age interval. Similar results have been found in other articles^18,21,30^.

Further, the multivariate logistic regression analysis performed in this study revealed age, education, smoking status and diabetes mellitus as significantly potential risk factors towards periodontitis.

Similarly to previous literature, periodontal complications was linked to aging within this population^12,18,21,31,32^. Moreover, the clinical periodontal hallmarks (CAL and PD), tooth loss and teeth with mobility were age-related. However, previous data and this survey suggest that intact supporting periodontal tissues prevail in patients of all age ranges, suggesting pathological CAL is not an aging consequence *per se*^32,33^.

Concerning the smoking status, being an active smoker was strongly associated with periodontitis (adjusted OR = 3.76), while past smoking history revealed a lower but also significant association (adjusted OR = 2.11). These results are in accordance with previous studies whose OR ranged between 2 and 6^14,18,34–36^ and is widely accepted that smoking has a harmful effect on the onset and progression of periodontitis along with other risk factors for periodontitis^13,37,38^. Likewise, it is also very important to highlight the influence of a past history of smoking activity and the repercussions of bad behaviours on the periodontal status and on tooth loss in the long-term^39^.

Regarding the education level, low educated people had a higher risk of having periodontitis being in line with previous reports^12,19,21,31,40^. In this population, the number of low educated participants is substantial and can be explained by the high number of elderly population and represent a generation that had little educational access. As a risk factor, low educational attainment has been linked to a greater loss of periodontal support and is more prominent when evaluated together with other sociocultural determinants^1,41,42^.

As well, DM was a risk factor towards periodontitis in this population in the same way as established in the literature^5,43,44^. Diabetes increases the risk for periodontitis (particularly if poorly controlled) and evidence suggests that advanced periodontitis also compromises glycaemic control. The new consensus has established DM as a grading modifier for the progression of periodontitis through the glycated hemoglobin (HbA1c) levels. Though, we have recorded HbA1c of our DM patients, as part of the standard clinical follow-up in FHUs, most non-DM patients have never been analyzed for, and to prevent bias in the multivariate analysis we will address this in a future focused study.

### Strengths and limitations

This survey has numerous strengths, including the representativeness and global geographic coverage based on the FHUs where the study was carried out, the sample size calculation stratified for each FHU, the strict methodology followed and the employment of the new AAP/EFP case definition enabling future comparability across studies.

Nevertheless, there are some shortcomings to mention. Due to the peculiarly low periodontitis prevalence previously reported and that based sample size calculation, more than half of the participants had ≥61 years old, which might have overestimated the prevalence of periodontitis. Also, the target population’s sociodemographic characteristics and oral hygiene behaviours must be carefully considered when extrapolating the present findings to other European populations, particularly the elderly subset that had low education and economic constraints. Lastly, people were directly invited to participate in the study, which can bias the population coverage for sampling, however also increases the probability of having a more accurate representation of the participant’s oral situation.

These findings provide new knowledge that will empower appropriate public oral health programmes and population-based preventive actions. Our results support that a comprehensive national oral program with higher accessibility is imperative. A national study is urgently needed to confirm the data reported in our investigation. Next, it will be essential to follow risk groups highlighted by the model developed in this study, namely the elderly, diabetics, smokers and those with low education. Finally, it is necessary to increase the population awareness to periodontitis signs, symptoms and consequences to the general health.

## Conclusions

This study reveals a high burden of periodontitis in the adult population of the southern region of the Lisbon Metropolitan Area, in Portugal. Age, education level, smoking status and diabetes mellitus were identified as significantly potential risk factors towards periodontitis.

### Methods

This study was approved by the Research Ethics Committee of the Regional Health Administration of Lisbon and Tagus Valley, IP (Portugal) (Approval numbers: 3525/CES/2018 and 8696/CES/2018) and in accordance with the Declaration of Helsinki, as revised in 2013. Following examination, each participant was informed of their periodontal status and signed informed consent prior to enrollment. Patients with diagnosed periodontal diseases were referred to the Egas Moniz Dental Clinic (EMDC) for treatment without additional costs. This survey followed the STrengthening the Reporting of OBservational studies in Epidemiology (STROBE) guidelines^45^.

### Study design and sampling procedure

The Study of Periodontal Health in Almada-Seixal (SoPHiAS) was designed as a population-based cross-sectional representative study, geographically stratified, with a target population of subjects over 18 years of age (adults and elderly), living in the municipalities of Almada and Seixal, in Portugal. Almada and Seixal, are two of the largest municipalities located in the southern part of the Lisbon Metropolitan Area, a NUTS II region (PT17). This region, with over 2.8 million inhabitants, includes 18 municipalities and is the most populated Portuguese Metropolitan Area and the second most populated NUTS II region of the country. In Portugal, all residents are covered by the National Health System and assigned to a General Practitioner of a public Family Health Unit (FHU). FHUs are grouped in Health Centers grouping (ACES), depending on the geographic region. For this study, the ACES Almada-Seixal was defined as the study group. All twenty-two ACES Almada-Seixal FHUs were included to ensure a global geographic and socioeconomic coverage of the Almada and Seixal territory. In September 2018, according to the institutional data provided, the two municipalities had 386,168 inhabitants in the selected age groups (adults and elderly). To achieve an estimate of the periodontitis prevalence in the population, with a margin of error of 3.0%, for a 95% confidence level, a minimum of 962 individuals were needed to be examined, based on the previously reported national prevalence data of 10.8% and 15.3%, for adults and elderly, respectively^15^. The required sample was stratified according to the number of adult (age group from 18–64 years) and elderly (65 years or older) subjects assigned to each FHU, based on the information provided by ACES Almada-Seixal. The invitation to participate in the survey was made by direct contact at the waiting room of the FHU, explaining the purpose of the study and including a description of the clinical examination. After a detailed explanation with the information sheet delivery to the patient, individuals who agreed to participate signed the informed consent form. A questionnaire was completed by each subject and collected before the periodontal examination.

### Gingivitis and periodontitis case definitions

Gingivitis and periodontitis cases were defined according to the new AAP/EFP consensus^13,23^. A gingivitis case was defined if total score of bleeding on probing (BoP) ≥10%^23^. A participant was a periodontitis case if: interdental CAL ≥2 non‐adjacent teeth, or Buccal or Oral CAL ≥3 mm with PD >3 mm is detectable at ≥2 teeth. Then, periodontitis staging was defined according to severity and extent^13^. For the severity, interdental CAL at site of greatest loss of 1–2 mm, 3–4 and ≥5 was considered as mild (stage 1), moderate (stage 2), and severe (stage 3 and stage 4), respectively^13^. Additionally, patients were defined for the extent, wherein a case was described as localized (<30% of teeth involved), generalized (≥30% of teeth involved) or molar/incisor pattern^13^.

### Clinical periodontal examination

Two calibrated investigators (VM and JB) performed a full-mouth periodontal examination, on an average of 30 minutes. Each clinical examination was performed under proper lighting with the individuals seated on a regular adjustable stretcher in the FHU’s medical office. No radiographic examination was made.

All fully erupted teeth, excluding third molars, implants and retained roots, were examined by means of a daily sterilized dental mirror and a manual periodontal North Carolina probe (Hu-Friedy; Chicago, Illinois, USA). The number of missing teeth was recorded. Further, dichotomous plaque index (PI), gingival recession (REC), PD, and BoP were circumferentially recorded at six sites per tooth (mesiobuccal, buccal, distobuccal, mesiolingual, lingual, and distolingual). PD was measured as the distance from the free gingival margin to the bottom of the pocket and REC as the distance from the cementoenamel junction (CEJ) to the free gingival margin, and this assessment was assigned a negative sign if the gingival margin was located coronally to the CEJ. CAL was calculated as the algebraic sum of REC and PD measurements for each site. The measurements were rounded to the lowest whole millimeter. Furcation involvement (FI) was assessed using a 2 N probe (Hu-Friedy; Chicago, Illinois, USA) following^46^ in molars, and upper first premolars if applicable, and tooth mobility was appraised following.

### Sociodemographic and medical questionnaires

Information on sociodemographic characteristics and behaviors was collected by self-reported questionnaire. The questionnaire covered questions on the following items: (1) gender, age, marital status, educational level, occupation; (2) monthly family gross income; (3) smoking habits; (4) oral hygiene-related behaviors (tooth brushing frequency, interproximal cleaning, etc.); (5) attitudes and awareness towards oral health; (6) diabetes mellitus (DM) and comorbidities^18^.

Education was categorized according to the 2011 International Standard Classification of Education (ISCED-2011)^47^: No education (ISCED 0 level), Elementary (ISCED 1–2 levels), Middle (ISCED 3–4 levels), Higher (ISCED 5–8 levels). Occupation status of each participant was classified as: student, employed, unemployed or retired. Marital status was defined as: married/union of fact, divorced, single or widowed. Smoking status was defined as non-smoker, current smoker or former smoker. Family gross income was categorised in three levels: less or equal to 600, 601 to 1500 and higher than 1500 euros per month.

### Measurement reliability and reproducibility

Two examiners (VM and JB) were trained under the supervision of an experienced senior periodontist (RA), prior to data collection. For the purpose of measurement reliability and reproducibility, a total of 10 volunteers seeking care at EMDC were randomly selected and evaluated. These patients were not further involved in the study. Volunteers were examined by the senior periodontist, the ‘reference examiner’, and the two field clinicians. Measurements were repeated one week later in the same volunteers. Measurement reliability and reproducibility were assessed by the intra-class correlation coefficient (ICC). Obtained ICC inter-examiner values were 0.98 and 0.99, for CAL and PD, respectively. The intra-examiner ICC ranged from 0.97 to 0.99, for both PD and CAL.

### Data analysis

Data analysis was performed using SPSS Statistics version 25.0 for Windows (IBM; Armonk, New York, USA). Descriptive and inferential statistics methodologies were applied. Spearman’s rank correlation coefficient (rho) was used to assess correlations between periodontal clinical data and age. Binomial logistic regression analysis was used to model the relationship between periodontitis and several potential risk factors. Preliminary analyses were performed using univariate models. Next, a multivariate model was constructed for periodontal disease estimation. Only variables showing a significance p ≤ 0.25 in the univariate model were included in the multivariate stepwise procedure. The contribution of each variable to the model was evaluated by Wald statistics. Interactions were also analyzed for all tested variables. The final reduced model was obtained with the following predictor variable categories: age, education, smoking status and diabetes. Odds ratio (OR) and 95% confidence intervals (95% CI) were calculated for both univariate and multivariate analyses. The level of statistical significance was set at 5% in all inferential analyses.

## Supplementary information

Supplementary File

## Data Availability

Due to legal arrangements with governmental institutions, we are not authorized to disclose data.
